# 

**Published:** 1901-06

**Authors:** 


### BOOKS RECEIVED.

Saunders's Medical Hand-Atlases. Atlas and Epitomé of the Nervous System and its Diseases. By Professor Dr. Chr. Jakob, of Erlangen. From the second revised German edition. Edited by Edward D. Fisher, M. D , Professor of Diseases of the Nervous System, University and Bellevue Medical College, New York. With 83 plates and copious text. Philadelphia and London: W. B. Saunders & Co. 1901. [Cloth, $3.50 net.]

Saunders's Medical Hand-Atlases. Atlas and Epitomé of Obstetric Diagnosis and Treatment. By Dr. O. Shaeffer, of Heidelberg. From the second revised German edition. Edited by J. Clifton Edgar, M. D., Professor of Obstetrics and Clinical Midwifery, Cornell University Medical School. With 122 colored figures on 56 plates, 38 other illustrations, and 317 pages of text. Philadelphia and London: W. B. Saunders & Co. 1901. [Cloth, $3.00 net.]

Saunders's Medical Hand-Atlases. Atlas and Epitomé of Labor and Operative Obstetrics. By Dr. O. Shaeffer, of Heidelberg. From the fifth revised German edition. Edited by J. Clifton Edgar, M. D., Professor of Obstetrics and Clinical Midwifery, Cornell University Medical School. With 14 lithographic plates, in colors, and 139 other illustrations. Philadelphia and London: W. B. Saunders & Co. 1901. [Cloth, $2.00 net.]

Saunders's Medical Hand-Atlases. Atlas and Epitomé of Ophthalmoscopy and Ophthalmoscopic Diagnosis. By Professor Dr. O. Haab, Director of the Eye Clinic in Zurich. From the third revised and enlarged German edition. Edited by George E. de Schweinitz, Professor of Ophthalmology, Jefferson Medical College, Philadelphia. With 152 colored lithographic illustrations and 85 pages of text. Philadelphia and London: W. B. Saunders & Co. 1901. [Price, $3.00 net.]

Saunders's Question Compends. Essentials of the Diseases of Children. By William M. Powell, M. D. Third edition. Thoroughly revised by Alfred Hand, Jr., M. D., Dispensary Physician and Pathologist to the Children's Hospital, Philadelphia. Duodecimo, 259 pages. Philadelphia and London: W. B. Saunders & Co. [Price, $1.00 net.]

A Text-Book of the Practice of Medicine. By Dr. Herman Eichhorst, Professor of Special Pathology and Therapeutics and Director of the Medical Clinic in the University of Zurich. Translated and edited by Augustus A. Eshner, M.D., Professor of Clinical Medicine in the Philadelphia Polyclinic. Two octavo volumes of over 600 pages each; over 150 illustrations. Philadelphia and London: W. B. Saunders & Co. 1901. [Price per set: Cloth, $6.00 net]

The Acute Contagious Diseases of Childhood. By Marcus P. Hatfield, A. M., M. D, Professor Emeritus of Diseases of Children, Northwestern University Medical School; Professor of Diseases of Children, Chicago Clinical School; Attending Physician, Wesley Hospital. Pages, 142. Chicago: G. P. Engelhard & Co. 1901. [Price, $1.00 net.]

A System of Physiologic Therapeutics. A Practical Exposition of the Methods, other than Drug-giving, useful in the Treatment of the Sick. Edited by Solomon Solis Cohen, A. M., M. D, Professor of Medicine and Therapeutics in the Philadelphia Polyclinic; Lecturer on Clinical Medicine at Jefferson Medical College; Physician to the Philadelphia and Rush Hospitals, etc. Volume I. Electrotherapy. By George W. Jacoby, M. D., Consulting Neurologist to the German Hospital, New York City; to the Infirmary for Women and Children; and to the Craig Colony for Epileptics, etc. In two books. Book I. Electrophysics—Apparatus Required for the Therapeutic and Diagnostic use of Electricity. Octavo, pp. 242; 163 illustrations. Philadelphia: P. Blakiston's Son & Co. 1901. [Price for the complete set, eleven volumes, $22.00.]

Uterine Fibromyomata. Their Pathology, Diagnosis and Treatment. By E. Stanmore Bishop, F.R.C.S. Eng. President Manchester Clinical Society; Fellow of the British Gynecological Society, etc. Octavo, pp. 323; 49 illustrations. Philadelphia: P. Blakiston's Son & Co. 1901. [Price, $3.50.]

3500 Questions on Medical Subjects. Arranged for self-examination. With the proper references to standard works in which the correct replies will be found. Third edition, enlarged. With questions of the state examining boards of New York, Pennsylvania and Illinois. Philadelphia: P. Blakiston's Son & Co. 1901 [Price, 10 cents.]

Clinical Pathology of the Blood. A treatise on the general principles and special applications of Hematology. By James Ewing, M. D., Professor of Pathology in Cornell University Medical College, New York City. In one handsome octavo volume of 432 pages, with 28 engravings and 14 full-page plates in colors. Just ready. Philadelphia and New York: Lea Brothers & Co. 1901. [Price, cloth, $3.50 net.]

A Reference Handbook of the Medical Sciences. Embracing the entire range of scientific and practical medicine and allied science By various writers. A new edition, completely revised and rewritten. Edited by Albert H. Buck, M. D., New York City. Volume II. Bla–Chl. Illustrated by numerous chromolithographs and 765 half-tone and wood engravings Nine volumes, imperial 8vo. New York: William Wood & Co. 1901. [Price, muslin, $6.00 per volume; leather, $7 00 per volume; half-morocco, $8.00 per volume.]

Contemporary Celebrities from the Album Mariani of Paris, France. New York: Published by Mariani & Co., 52 West 15th Street. 1901.

Whitman's Orthopedic Surgery. For students, practitioners and specialists. By Royal Whitman, M. D., Adjunct Professor of Orthopedic Surgery, New York Polyclinic; Instructor in Orthopedic Surgery in the College of Physicians and Surgeons, and Chief of Orthopedic Department in the Vanderbilt Clinic, New York. In one handsome octavo volume of 642 pages, with 447 illustrations. Philadelphia and New York: Lea Brothers & Co. 1901. [Price, cloth, $5.50 net.]

The Diagnosis and Treatment of Diseases of the Nose, Throat, Naso-Pharynx and Trachea. For the use of students and practitioners. By Cornelius G. Coakley, M. D., Professor of Laryngology in the University and Bellevue Hospital Medical College, New York. New (second) edition. In one handsome 12mo volume of 556 pages with 103 engravings and 4 colored plates. Philadelphia and New York: Lea Brothers & Co. 1901. [Cloth, $2.75 net.]

A Practical Treatise on Simple and Chronic Specific Urethritis By J. Henry Dowd, M. D., late Genito-Urinary Surgeon at the Buffalo Hospital of the Sisters of Charity; formerly Chairman of the Surgical Section of the Buffalo Academy of Medicine Illustrated 16mo, pp. xxxi.—135. Buffalo: A. W. Landsettle. 1901. [Price, $1.50.]

